# Draft Genome Sequence of Medium-Chain-Length Polyhydroxyalkanoate-Producing *Pseudomonas putida* Strain LS46

**DOI:** 10.1128/genomeA.00151-13

**Published:** 2013-04-18

**Authors:** Parveen K. Sharma, Jilagamazhi Fu, Xiangli Zhang, Brian W. Fristensky, Karen Davenport, Patrick S. G. Chain, Richard Sparling, David B. Levin

**Affiliations:** Department of Biosystems Engineering, University of Manitoba, Winnipeg, MB, Canadaa;; Department of Plant Science, University of Manitoba, Winnipeg, MB, Canadab;; Department of Microbiology, University of Manitoba, Winnipeg, MB, Canadac;; Bioscience Division, Los Alamos National Laboratory, Los Alamos, New Mexico, USAd

## Abstract

We describe the draft genome sequence of *Pseudomonas putida* strain LS46, a novel isolate that synthesizes medium-chain-length polyhydroxyalkanoates. The draft genome of *P. putida* LS46 consists of approximately 5.86 million bp, with a G+C content of 61.69%. A total of 5,316 annotated genes and 5,219 coding sequences (CDS) were identified.

## GENOME ANNOUNCEMENT

The genus *Pseudomonas* is comprised of a heterogeneous group of Gram-negative bacteria with diverse metabolic potential that reflects the different environments from which these species were isolated (1). They belong to the class *Gammaproteobacteria*, and to date, the *Pseudomonas* genome database lists 132 completed or draft genome sequences (2, 3). *Pseudomonas putida* strains have versatile metabolic capacity and have been developed as biocontrol agents for plant diseases, as bioremediation agents, and as commercial strains for biopolymer production (4, 5).

Here we report the genome sequence of *P. putida* strain LS46, which was isolated by enrichment from wastewater on the basis of its ability to synthesize medium-chain-length polyhydroxyalkanoates (mcl-PHAs). *P. putida* LS46 can utilize glucose, glycerol (including biodiesel-derived glycerol), fatty acids, vegetable oils, and waste fryer oils as carbon sources and synthesizes mcl-PHAs consisting of 3-hydroxy fatty acids with 6 to 14 carbon atoms (6).

The sequencing of *P. putida* strain LS46 was performed at McGill University, Montreal, Quebec, Canada, using a combination of Illumina Gaii (7) and 454 (8) technologies. The draft assembly was generated at the Genome Science Group, Bioscience Division, Los Alamos National Laboratory, Los Alamos, NM, combining the Illumina shotgun library, which contained 310,429,960 reads, and a paired-end 454 library with an average insert size of 8 kb, which contained 230,815 reads totaling 85 Mb. An estimated 300-fold coverage of Illumina data was assembled with VELVET, version 1.1.05 (9), and the consensus sequences were computationally shredded into 1.5-kb overlapping sequences. These 1.5-kb sequences and the estimated 15-fold coverage of 454 paired-end data were assembled together with Newbler, version 2.6. The Newbler consensus sequences were computationally shredded into 2-kb overlapping fake reads (shreds). All data were additionally assembled together with Allpaths, version 39750, and the consensus sequence was computationally shredded into 10-kb overlapping fake reads (shreds). We integrated the 454 Newbler consensus shreds, the Illumina VELVET consensus shreds, Allpaths consensus shreds, and the read-pairs in the 454 paired-end library using parallel Phrap, version SPS-4.24 (High Performance Software, LLC). Possible misassemblies and gaps were resolved using a series of Perl and Java scripts, combined with manual editing in Consed (10, 11, 12). The total size of the high-quality draft genome sequence is 5.8 Mb.

The genome of *P. putida* LS46 consists of 5,862,556 bp with a G+C content of 61.69%. A total of 5,316 genes and 5,219 coding sequences (CDS) were present. Among RNA genes, 23 rRNA genes (8 5S, 7 16S, and 8 23S) and 74 tRNA genes were identified. The genome sequence of *P. putida* LS46 is similar to the nine genome sequences of *P. putida* strains (KT2440, F1, GB-1, W619, BIRD-1, S16, ND6, UW4, and DOT-T1E) available at the Joint Genome Institute website (http://img.jgi.doe.gov), but the *P. putida* LS46 genome has unique features which differentiate it from the genomes of these other strains (1).

### Nucleotide sequence accession number. 

The genome sequence of *P. putida* LS46 has been deposited in the GenBank database under accession number ALPV00000000. The version described in this paper is the first version.
